# Zinc and metallothionein dysregulation in B cells in multiple sclerosis, with disease-specific CXCR3^+^/CXCR3⁻ divergence in blood

**DOI:** 10.3389/fimmu.2026.1922905

**Published:** 2026-08-28

**Authors:** Remi Fritzen

**Affiliations:** 1 School of Physics and Astronomy, University of St Andrews, St Andrews, United Kingdom; 2 Centre of Biophotonics, University of St Andrews, St Andrews, United Kingdom

**Keywords:** cerebrospinal fluid, CXCR3^+^ B cells, multiple sclerosis, single-cell RNA sequencing, zinc homeostasis

## Abstract

**Introduction:**

Multiple sclerosis (MS) is associated with compartmentalized B-cell responses in the cerebrospinal fluid (CSF), where CXCR3^+^ B cells contribute to intrathecal immune activity and tissue injury. Zinc is an important regulator of immune-cell function, but zinc homeostasis in pathogenic B-cell populations in MS remains poorly defined.

**Methods:**

We reanalyzed two publicly available human single-cell RNA sequencing datasets containing CSF and peripheral blood (PB) B cells from healthy or non-MS controls and patients with clinically isolated syndrome or MS. Zinc transporter and metallothionein (Zn/MT) gene expression was examined in CXCR3^+^ and CXCR3^−^ B-cell subsets, and a composite Zn/MT signature was calculated at the patient level.

**Results:**

A composite Zn/MT signature was significantly elevated in MS relative to controls in both PB and CSF, and in both CXCR3^+^ and CXCR3^−^ B cells, indicating a disease-associated feature of B cells broadly rather than one restricted to CXCR3^+^ cells. In CSF CXCR3^+^ B cells, this increase was driven by several zinc transporters, including *SLC39A11* (ZIP11), *SLC30A9* (ZnT9), and *SLC39A6* (ZIP6), whereas no individual gene reached significance in PB. Direct comparison of CXCR3^+^ and CXCR3^−^ B cells revealed a disease-specific divergence in PB, with a significantly higher Zn/MT signature in CXCR3^+^ cells from MS patients but not healthy controls. Five genes contributed to this effect.

**Discussion:**

These findings identify altered zinc-homeostasis pathways as a disease-associated feature of B cells in MS and reveal a disease-specific divergence between CXCR3^+^ and CXCR3^−^ B-cell subsets in peripheral blood. The results support further investigation of zinc-regulatory mechanisms in B cell-mediated neuroinflammation.

## 1 Introduction

Multiple sclerosis (MS) is a chronic inflammatory neurodegenerative disease and a leading cause of non-traumatic disability in young adults (1). MS is characterized by loss of integrity of the blood–brain barrier (BBB) (2) and consequent accumulation of immune cells in the cerebrospinal fluid (CSF). Multiple independent studies have shown that CXCR3-expressing, T-bet–high B cells are enriched in the CSF and CNS compartments of MS patients, display clonal evolution from memory B cells to plasmablasts, and amplify Th1-skewed inflammation, while circulating CXCR3^+^ memory B cells exhibit CNS-homing features and a propensity to become antibody-secreting cells (3–6). Consistent with this, CXCL10, a key CXCR3 ligand, is elevated in MS CSF and associates with increased CSF immune cell infiltrates and longer disease duration (7).

Zinc is an essential trace element involved in many biological processes, including B cell maturation and function (8, 9), and differentiation of regulatory B cells (10). Several studies have linked altered zinc status to MS, although with mixed results (11–13). Moreover, trace elements analysis in CIS implicates deficiencies in multiple metal ions, including zinc, in a first demyelinating event (14). However, zinc “deficiency” does not capture the full picture: abnormal spatial or compartmental distribution of zinc might influence MS pathogenesis even transiently, while being invisible to measurements that focus solely on systemic zinc deficiency (11).

Zinc concentration differs markedly between plasma and CSF, with CSF containing 10 to 15% of the concentration found in plasma (15). When immune cells enter the CSF, this change in their environment may therefore have a profound impact on their behavior, including their pathogenic potential, but this question has not been systematically addressed in MS. On this basis, we hypothesized that a distinct zinc signature might be present in CXCR3+ B cells in MS as cells are exposed to the low-zinc environment of the CSF. To test this, we reanalyzed existing single-cell RNA-seq datasets comparing zinc homeostasis-related gene expression (including transporters and metallothionein (MTs)) in different compartments, blood and CSF in MS and healthy and non-MS controls (subsequently termed “healthy controls” (HC)) and between CXCR3^+^ and CXCR3^-^ B cell subsets. The data show that a zinc/metallothionein signature is elevated in MS relative to HC in CXCR3^+^ B cells in both blood and CSF, with individual transporter genes, including ZIP11, ZnT9, and ZIP6, reaching significance specifically in CSF, consistent with a stress-associated zinc-handling response (16, 17). However, we found no evidence that this signature is induced locally by the CSF environment: neither individual genes nor the composite signature differed between PB and CSF within CXCR3^+^ B cells, in either HC or MS, suggesting the signature is established during peripheral activation rather than upon CNS entry. These observations support a model in which zinc-handling is a disease-associated feature of the CXCR3+ effector subset established in the periphery, rather than one driven by local zinc scarcity in the CSF itself.

## 2 Materials and methods

### 2.1 Sex as a biological variable

Both female and male participants were represented in the public human datasets analyzed in this study. Female participants comprised 58% of GSE138266 and 69% of GSE133028 (**Supplementary Table 1**). Given known sex-related differences in zinc physiology, we performed a sex-stratified sensitivity analysis within MS patients to assess whether our primary findings were confounded by sex composition (see Results).

### 2.2 Datasets and cell selection

Single-cell datasets containing peripheral blood and cerebrospinal fluid B cells were integrated from previously published studies, and analyses were restricted to cells expressing B cell markers (*CD19*, *MS4A1*, *CD79A*, *CD79B*, *CD74*, *IGHM*, *IGHD*, *IGKC*, *IGLC2*) after batch integration and removal of non-B cell contaminants. B cells were identified using a two-step gate: Cells expressing any of the above markers (raw count >0) were retained, followed by exclusion of cells with high expression of non b cell markers (T/NK: *CD3D*, *CD3E*, *TRAC*, *TRBC1*, *TRBC2*, *NKG7*, *GNLY*, *PRF1*, monocyte and myeloid cells *LST1*, *LILRB1*, *LILRB2*, *S100A8*, *S100A9*, *LYZ*, *FCGR3A*, *C1QA*, *C1QB*, *C1QC*, erythroid and megakaryocyte: *HBB*, *HBA1*, *HBA2*, *PF4, PPBP*). Cells in the top 30% of mean non-B marker expression among B-marker positive cells were excluded as likely contaminant or doublets. CXCR3-positive analyses were performed on cells labelled as CXCR3^+^ and belonging to either the healthy control or MS groups within each tissue compartment. The distribution of CXCR3 expression across datasets did not allow for discrimination between two clear population therefore cells were considered positive when CXCR3 was detectable (**Supplementary Figures 1A, B**), UMI > 0 (18).

Sequencing depth and quality were comparable between PB and CSF B cells, with similar total counts and mitochondrial percentages per cell across tissues (**Supplementary Figures 1C, D**). As expected, PB yielded more B cells than CSF (**Supplementary Figure 1E**), leading to unequal cell numbers for PB versus CSF comparisons. We therefore used non-parametric tests for signature comparisons and interpret CSF-restricted effects with attention to sample size.

### 2.3 Zinc-related gene set and signature

A curated zinc-related gene set was assembled from zinc transporter genes and MTs after excluding biologically irrelevant genes for B cells (*i.e* ZnT2 (only expressed mammary and intestinal cells), ZnT8 (only expressed in pancreatic Beta cells), Znt3 (only expressed in neurons)). Metallothioneins genes that were expressed in less than 1% of cells in CXCR3^+^ B cells were not included (*MT1A*, *MT1G*, *MT1H*, *MT1M*) (**Supplementary Table 2**). For each CXCR3^+^ B cell, 2 zinc signatures were calculated the first one related to the analysis of disease related differences, includes *a priori* direction of change to avoid a pure mean effect where expression levels of transporters and MTs would tend to cancel out, MT expression were multiplied by -1 and zinc transporters stayed as is. This is driven by the knowledge that cells in disease states over express zinc transporters and limit the expression of buffering proteins MT. The second signature was expressed as the mean expression across the curated Zn/MT gene set present in the expression matrix.

Because this signature is derived from the processed single-cell expression matrix, its absolute values can be either negative or positive depending on the upstream normalization and centering of the data. A value below zero therefore does not indicate biologically negative zinc abundance; instead, the signature should be interpreted relatively, such that higher values indicate higher expression of the zinc-related transcriptional program within the analyzed dataset.

### 2.4 Statistics

All statistical analyses were performed in Python 3.12.11 (numpy 2.4.6, pandas 3.0.5, scipy 1.18.0, scanpy 1.12.1, anndata 0.12.16, statsmodels 0.14.6, harmonypy 2.0.0, matplotlib 3.10.9, seaborn 0.13.2). Because single cells from the same patient are not statistically independent, all group comparisons were performed on patient-level pseudobulk values (mean expression per patient across the relevant cell subset) rather than on individual cells, to avoid pseudoreplication. Comparisons between independent groups, where each patient belongs to a single condition (MS vs. HC), used a two-sided Mann–Whitney U test; comparisons where the same patient contributed to both arms (CXCR3^+^ vs. CXCR3⁻; PB vs. CSF) used a two-sided Wilcoxon signed-rank test on each patient’s own paired difference. Benjamini–Hochberg correction was applied genome-wide (all genes passing expression-prevalence filtering) and, separately, restricted to the pre-specified 14-gene Zn/MT panel; both are reported where relevant. Patient-level associations between the Zn/MT signature and EDSS were assessed using Spearman correlation, with leave-one-out robustness checks for any correlation surviving correction. Dataset-of-origin confounding was assessed for all pooled analyses by stratified testing within each source dataset. Full detail for all statistical procedures, including the competitive gene-set rank test and dataset-confounding checks, is provided in **Supplementary Methods**. P values below 0.05 were considered statistically significant after the correction described above.

### 2.5 Study approval

This study is a secondary analysis of de-identified, publicly available single-cell RNA-seq datasets (GSE138266, GSE133028) and did not involve new recruitment of human participants. Institutional review board approval and written informed consent were obtained in the original studies, as described in their respective publications.

## 3 Results

### 3.1 Datasets and harmonization

**Figure 1:**
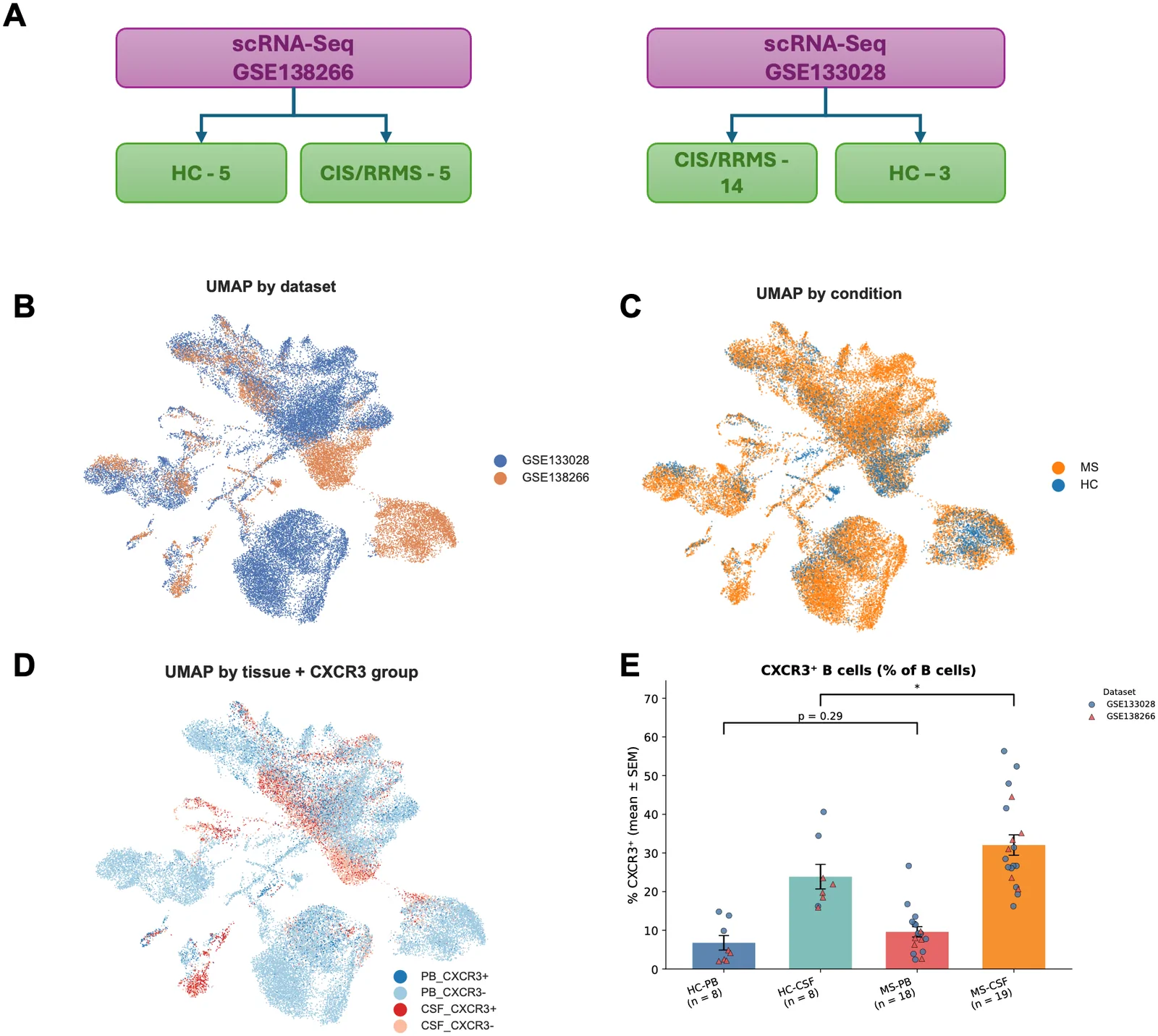
Integrated CSF and blood B cell datasets and CXCR3^+^ compartment. **(A)** Overview of the two scRNA-seq datasets (GSE138266, GSE133028) and patient numbers per diagnostic group. Only green groups were kept. **(B)** UMAP of harmonized B cells colored by dataset, showing good overlap between studies. **(C)** UMAP colored by condition (HC and MS), indicating mixing of diagnostic groups across B-cell states. **(D)** UMAP colored by tissue and CXCR3 status (PB/CSF, CXCR3^+^/CXCR3⁻), illustrating relative enrichment of CXCR3^+^ cells in CSF. **(E)** Frequencies of CXCR3^+^ B cells among total B cells in PB and CSF across groups, with higher proportions in CSF and a trend towards increase in demyelinating disease (p-values from two-sided tests as shown, *p=0.049).

We reanalyzed two scRNA datasets containing cells from both cerebrospinal fluid (CSF) and peripheral blood (PB) (GSE138266 (19), GSE133028 (3)) from MS patients and non-MS patient or healthy controls (grouped under the umbrella term: HC) (**Figure 1A**). While the metadata of GSE133028 allows for discrimination between CIS and MS, GSE138266 does not. Therefore, for the purpose of this study, MS and CIS patients were collated. Steroid-treated MS as well as uveitis, neurosarcoidosis and neuromyelitis patients in GSE133028 were excluded. The datasets were harmonized and cells expressing non-B cell markers were filtered out (**Supplementary Table 1**).

Conditions (HC and MS) as well as CXCR3^-^ and CXCR3^+^ cells in different compartments are shown (**Figures 1C, D**): different B cell subsets contained CXCR3-expressing cells in both CSF and PB. CXCR3^+^ B cells were more frequent in CSF than in PB (~30% and ~14%, respectively; **Figure 1E**). We also found a borderline-significant difference in the proportion of CXCR3^+^ B cells between HC and MS patients (24% ± 9% *vs*. 32% ± 12%, respectively; p = 0.049).

### 3.2 Zinc–metallothionein signature in CXCR3^+^ B cells

**Figure 2:**
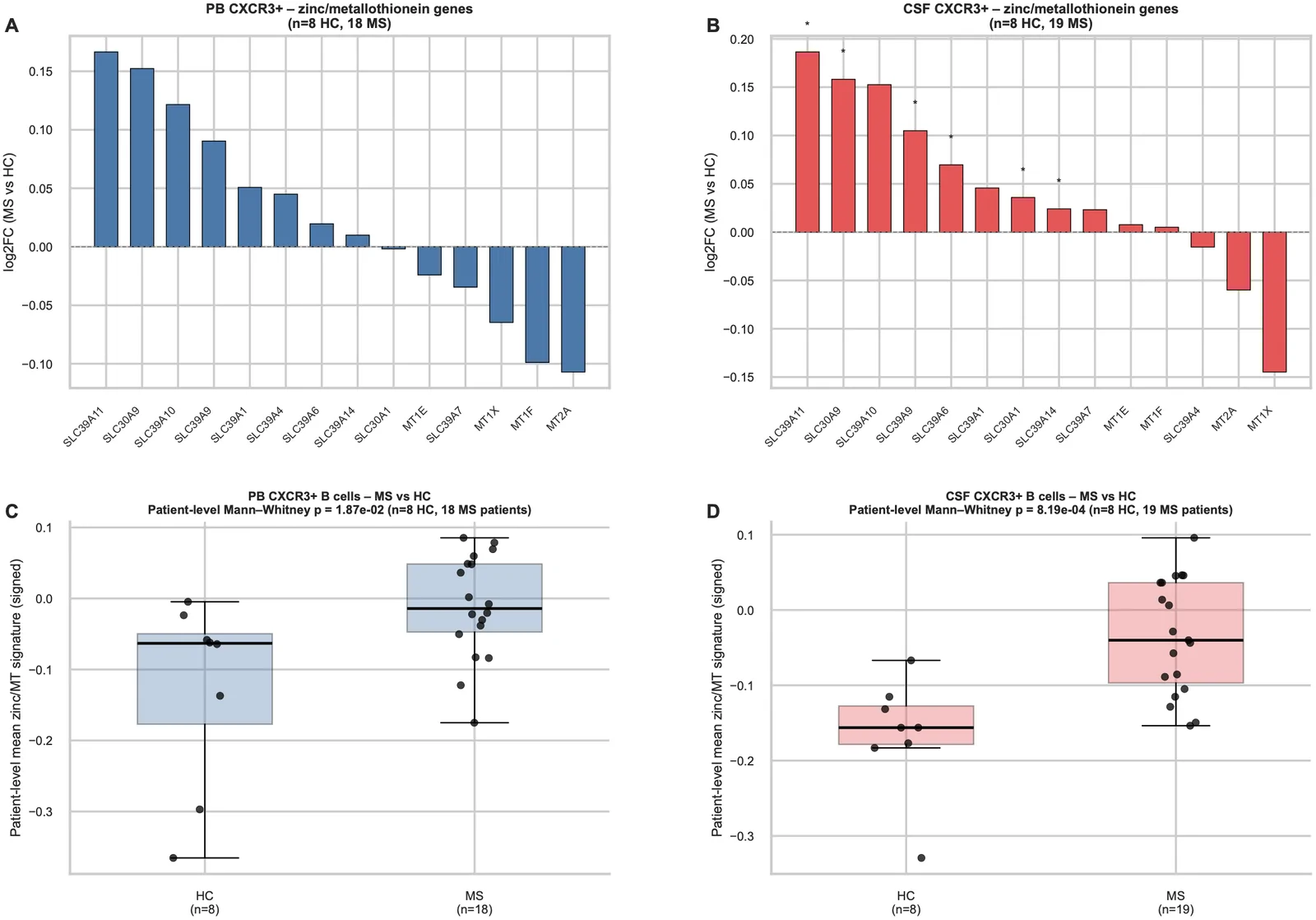
Zn/MT genes and signature in CXCR3^+^ B cells from PB and CSF. **(A)** Log2 fold-change (MS vs healthy controls) of zinc transporter and MT genes in peripheral blood (PB) CXCR3^+^ B cells, highlighting upregulation of *SLC39A11* and several zinc transporters in MS. **(B)** Log2 fold-change (MS vs healthy controls) of Zn/MT genes in cerebrospinal fluid (CSF) CXCR3^+^ B cells, with multiple transporters increased and MTs reduced in MS. Violin plots of Zn/MT signature in **(C)** PB and **(D)** CSF CXCR3^+^ B cells. The statistical test is made on a per-patient average of signature zinc values in MS and HC in PB (Mann–Whitney p ≈ 1.87.10⁻^2^) and CSF (Mann–Whitney p ≈ 8.19.10⁻^4^).

Following the global characterization of these mixed datasets, zinc-related genes were chosen. These included MTs, zinc transporters SLC30Axx (ZnT) and SLC39Axx (ZIP) which import and export zinc to and from the cytoplasm. Additionally, genes known not to be expressed in B cells (such as Znt8 or ZnT3), were excluded. We hypothesized that zinc transporters will generally be upregulated and MTs be downregulated as inflammation drives increase in zinc uptake and zinc availability might need to increase. We calculated expression levels of each of these genes in CXCR3+ per patient and compared this between MS and HC in both PB (**Figure 2A**; **Supplementary Figure 2A**) and CSF (**Figure 2B**; **Supplementary Figure 2B**). In the PB, no difference in gene expression was significant, which might be due to a low n number (n_HC_: 8, n_MS_: 19), however, the pattern was as mainly hypothesized (higher expression of transporters, lower expression of MTs). In the CSF most transporter genes reached statistical significance (**Supplementary Table 3**), showing a similar pattern consistent with our hypothesis.

To assess this further, we calculated a per-patient signed zinc signature by averaging the expression of all genes in the set for each patient, to avoid cancellation of up and downregulated genes, MTs expression data were multiplied by -1 (signed). Comparing this signature between HC and MS patients revealed significantly higher gene expression in CXCR3^+^ B cells in MS. This was observed in both peripheral blood (PB) and cerebrospinal fluid (CSF) as shown in **Figures 2C, D** respectively. The Mann-Whitney test confirmed this finding with p-values of 1.87.10^−2^ in PB and 8.19.10^−4^ in CSF. Volcano plots are shown in **Supplementary Figures 2A, B** as well as unsigned zinc/MT signature plots for PB and CSF in **Supplementary Figures 2C, D** respectively.

To assess whether this signature was confounded by sex, we compared male and female MS patients directly within this population, using the same patient-level pseudobulk framework. The composite signature did not differ significantly by sex in either PB (n=6 male, 12 female; p=0.335) or CSF (n=7 male, 12 female; p=0.642), nor did any of the six genes individually significant between MS and HC (all p_adj_ > 0.05 by sex). Healthy controls (n=8) were not included in this analysis, as splitting this group by sex would yield strata too small (~3–4 per group) to test reliably.

We performed the same analysis on CXCR3⁻ B cells, although this population is more heterogenous. The patient-level analysis and signed zinc signature showed a similar pattern, with significantly upregulated transporters while MTs are downregulated in both PB (**Supplementary Figure 3A**) and CSF (**Supplementary Figure 3B**; **Supplementary Table 4**). Consistent with this, the composite zinc/MT signature was elevated for MS compared to HC in PB and CSF (**Supplementary Figures 3C, D**) Mann–Whitney p = 4.16.10^−3^ and 5.55.10^−4^ respectively).

### 3.3 Zinc homeostasis genes are less variable than the background variation

We compared CXCR3^+^ B cells from PB *vs.* CSF to assess whether zinc and MT gene regulation differed between these compartments. In HC and MS, no genes were significantly differentially expressed, and the unsigned zinc signature was not different. It has to be acknowledged here that the detected differences were subtle and if true, our statistical analysis is not able to confirm them, in part due to the number of samples available as well as the low number of cells in that compartment (**Supplementary Table 1**, **Supplementary Figure 4**). In contrast to CXCR3^+^ B cells, where no individual gene reached significance for the PB-versus-CSF comparison, CXCR3⁻ B cells from MS patients showed a coordinated shift in Zn/MT gene expression between compartments. Seven of fourteen curated genes were significantly differentially expressed (Benjamini-Hochberg (BH)-corrected within the curated panel, p_adj_ < 0.05; **Supplementary Table 5**) but showed a mixed pattern among the transporters (**Supplementary Figure 5**). This MT-dominated, largely transporter-independent shift is the reverse of the transporter-up/MT-down pattern associated with disease in CXCR3^+^ cells (**Figure 2**) and was not observed in HC CXCR3⁻ B cells, where no gene survived correction (0/14; **Supplementary Figures 5C, D**). Paired patient-level trajectories (**Supplementary Figures 5E, F**) were heterogeneous, with individual patients moving in both directions between compartments.

**Figure 3:**
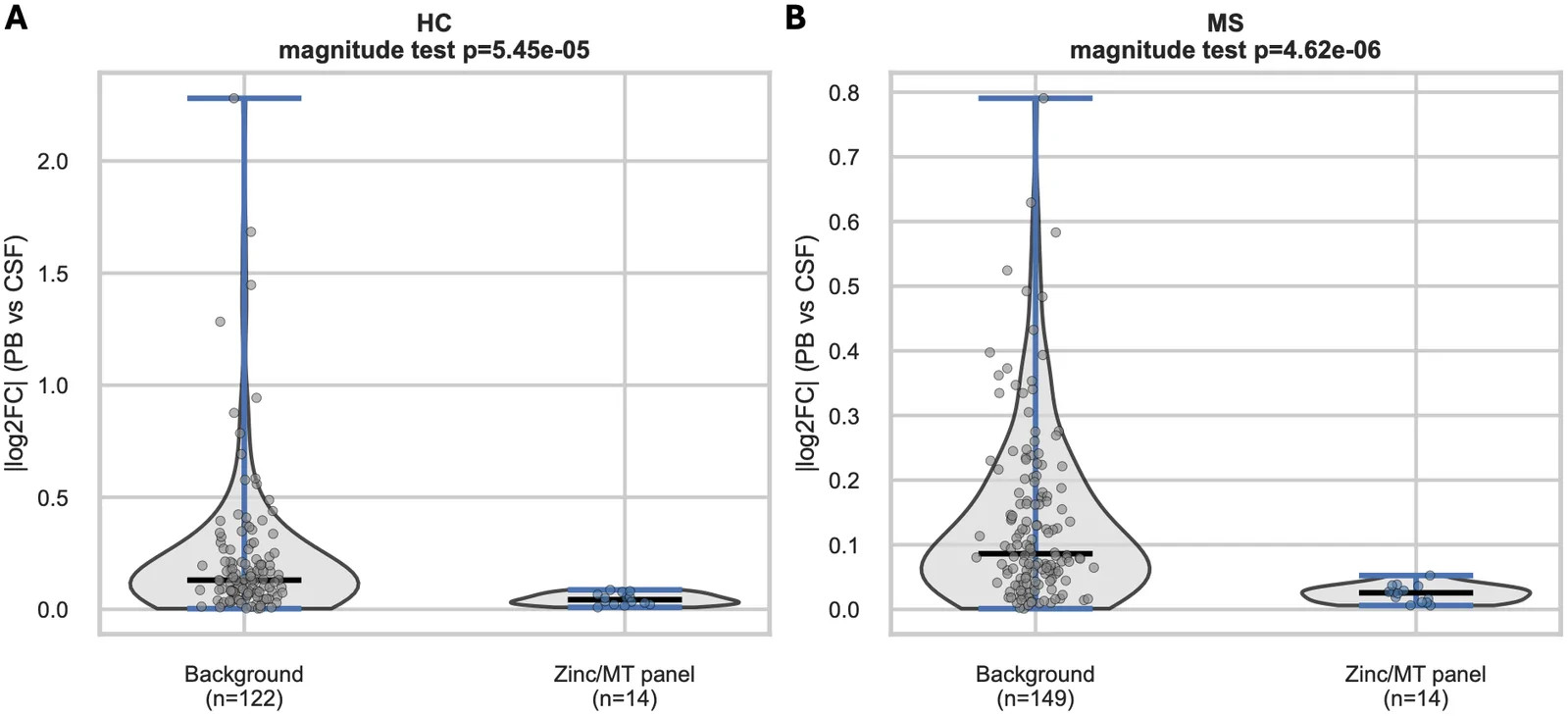
The curated Zn/MT panel varies less across the PB-CSF transition than background genes, in CXCR3^+^ B cells. Violin plots of absolute log2 fold-change (|log2FC|, PB vs. CSF) for background genes (grey, all other tested genes) versus the curated 14-gene Zn/MT panel (blue), in CXCR3^+^ B cells from **(A)** HC and **(B)** MS patients; individual genes shown as jittered points. The Zn/MT panel showed significantly smaller |log2FC| than background genes in both groups (competitive, direction-agnostic magnitude test, two-sided Mann-Whitney U; HC: median |log2FC| panel vs. background, p = 5.45.10⁻^5^; MS: 0.026 vs. 0.086, p = 4.62.10⁻^6^), indicating this gene set is under tighter regulatory constraint across the PB-CSF transition than the typical gene, independent of disease state.

Given the limited number of CXCR3^+^ cells available per patient for pseudobulk aggregation, and the absence of significant findings at the level of individual genes or the composite signature (above), we applied a competitive gene-set rank test to ask whether the curated Zn/MT panel behaved differently from the broader transcriptome across the PB-to-CSF transition, independent of any single gene reaching significance. A directional (location) test showed a modest, uncorrected shift toward higher (less negative) log2FC in the zinc/MT panel relative to background genes in both HC (median log2FC: panel = −0.032, background = −0.086; p = 0.053) and MS (panel = +0.006, background = −0.048; p = 0.048) (**Figure 3**), consistent with relative preservation of zinc/MT expression against a broader compositional decline across compartments rather than absolute upregulation. A complementary, direction-agnostic magnitude test showed that the zinc/MT panel varied significantly less than background genes in both groups (HC: p = 5.45 × 10^-5^; MS: median |log2FC| = 0.026 *vs*. 0.086 for background, p = 4.62 × 10^-6^), indicating that this gene set is subject to tighter regulatory constraint across the PB-CSF transition than the typical gene. Because this pattern was of similar magnitude in HC and MS, we interpret it as a general feature of Zn/MT gene regulation rather than a disease-specific effect.

We then compared CXCR3^+^ to CXCR3⁻ B cells within each tissue compartment to assess whether a differentially expressed zinc program could be detected specifically in the CXCR3^+^ effector subset, using a paired, patient-level framework (**Supplementary Figure 6**, PB; **Supplementary Figure 7**, CSF). In PB, five of fourteen curated genes were significantly higher in CXCR3^+^ relative to CXCR3⁻ B cells in MS patients (BH-corrected within the curated panel, p_adj_ < 0.05): *MT2A* (log2FC ≈ 0.19), *MT1X* (log2FC ≈ 0.13), *MT1F* (log2FC ≈ 0.07), *MT1E* (log2FC ≈ 0.07), and *SLC39A7* (log2FC ≈ 0.05), consistent with a zinc-programmed CXCR3^+^ subset in blood. This was reflected at the patient level, where the composite Zn/MT signature was significantly higher in CXCR3^+^ than CXCR3⁻ B cells in MS (paired Wilcoxon signed-rank p = 0.016, n = 18 patients) but not in HC (p = 0.383, n = 8), indicating a disease-specific effect. In contrast, this pattern was largely absent in CSF: individual genes showed little to no significant difference between CXCR3^+^ and CXCR3⁻ B cells after correction, and the composite signature did not differ significantly in either MS (p = 0.717, n = 19) or HC (p = 0.547, n = 8). *SLC39A11* (ZIP11), which distinguished MS from HC within CXCR3^+^ B cells (**Figure 2**), did not distinguish CXCR3^+^ from CXCR3⁻ B cells in either compartment, suggesting it marks disease status broadly rather than the CXCR3^+^ effector phenotype specifically. Together, these results indicate that the CXCR3^+^ zinc-programming signature is most evident in peripheral blood, developed in a disease-specific manner, rather than being a uniform feature of CXCR3^+^ B cells across compartments.

### 3.4 Patient-level zinc signature and expanded disability status scale (EDSS)

**Figure 4:**
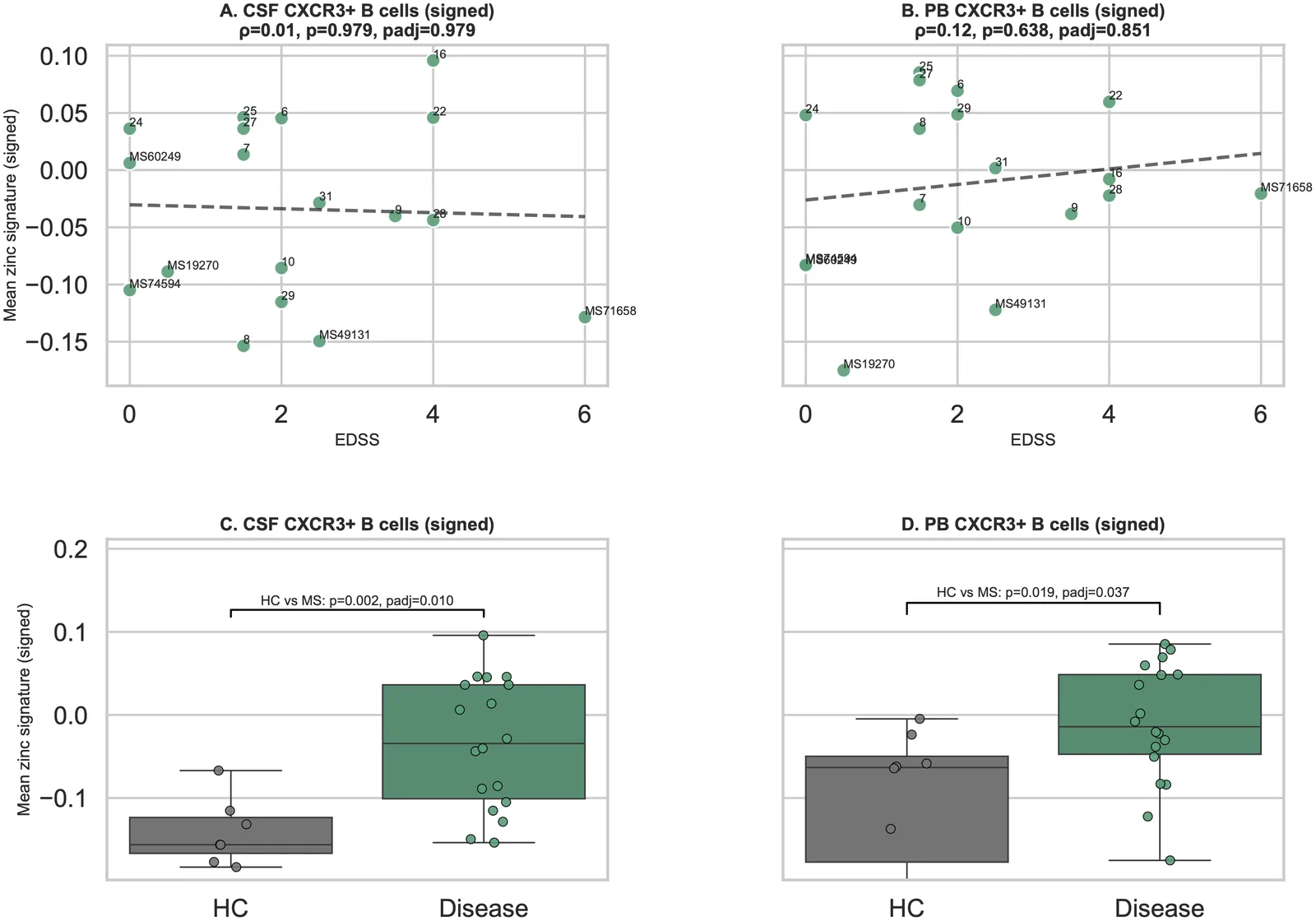
Zn/MT signature in B cells by disability and disease status. **(A, B)** Mean Zn/MT signature per patient plotted against Expanded Disability Status Scale (EDSS) for CSF **(A)** and peripheral blood (PB) **(B)**, dashed lines show that linear fits and Spearman correlations are weak and non-significant (ρ = 0.01, p_adj_ = 0.979 for CSF; ρ = 0.12, p_adj_ = 0.851 for PB). **(C, D)** Distribution of mean Zn/MT signature in CSF **(C)** and PB **(D)** B cells from HC versus patients with MS, showing a significant higher signature in CSF (p_adj_ = 0.010) and a and in PB (p_adj_ = 0.037; two-sided Mann–Whitney tests).

At the patient level, the sign-corrected composite Zn/MT signature showed no significant association with EDSS in either compartment (CSF: ρ = 0.01, p = 0.979, p_adj_ = 0.979; PB: ρ = 0.12, p = 0.638, p_adj_ = 0.851; **Figures 4A, B**). In contrast, when patients were grouped categorically by disease status, the signature was significantly higher in MS relative to HC in both compartments, surviving Benjamini–Hochberg correction across all four panels (CSF: p_adj_ = 0.010; PB: p_adj_ = 0.037; **Figures 4C, D**). These results indicate that the Zn/MT signature distinguishes MS patients from healthy controls categorically but does not track continuously with graded disability as measured by EDSS in this cohort. Finally, the association was tested for each gene individually (**Supplementary Figure 8**), although only SLC39A7 showed a significant correlation (ρ = 0.71, p_adj_ = 0.021) between EDSS and differential expression level.

## 4 Discussion

Here we show that a composite Zn/MT signature is significantly elevated in MS relative to HC at the patient level, in across PB and CSF and in both CXCR3^+^ and CXCR3^-^ B cells, indicating that this signature reflects a disease-associated feature of B cells broadly, rather than a phenomenon unique to the CXCR3^+^ effector subset. These findings extend recent work (6, 20) defining TBX21^+^CD21_lo_CXCR3^+^ B cells as an early, pathogenic B-cell population in MS by adding zinc-handling as a linked transcriptional module across B cells generally, with a specific disease associated divergence between CXCR3^+^ and CXCR3^-^ subsets described below. In this framework, altered zinc transport in B cells, and specifically its divergence between CXCR3^+^ and CXCR3^-^ subsets, may contribute to effector programming providing a plausible mechanistic link between zinc homeostasis and CNS-directed autoimmunity.

B cells are at the center of MS pathogenesis. Zinc homeostasis has been shown to play a role in B cell maturation and differentiation. Furthermore, zinc concentration is markedly lower in the CSF than in the peripheral blood, and CXCR3^+^ B cells are thought to traffic to the CNS through the interaction with CXCR3 ligands, particularly CXCL10 which has been implicated in lymphocyte recruitment during CNS inflammation (4). The presence of CXCR3^+^ B cells in the brain has been linked to brain-homing, T-bet–expressing B-cell populations that accumulate in the CNS and contribute to early MS activity and tissue injury (20). This led us to hypothesize that these cells would need to modify zinc-related gene expression to 1/adapt to the lower zinc concentration, and 2/sustain a pathogenic behavior. The fold change in expression was not expected to be high as zinc transporter and MT expression is tightly regulated and small change in their expression can have significant impact (21, 22).

Reanalyzing publicly available datasets, we show that several zinc-related genes are significantly altered in both CXCR3^+^ and CXCR3^-^ B cells from MS patients compared with HC. Notably, no genes were found to be differentially expressed in PB, in CXCR3^+^ B cells (despite a significant composite signature there) but several were in the CSF. For instance, *SLC39A11* (ZIP11), and *SLC39A6* (ZIP6) are over-expressed. Other over-expressed transporters include ZnT9, a conserved mitochondrial zinc transporter important for embryonic development. No MT genes individually reached significance in the CXCR3^+^ MS *vs.* HC comparison in either tissue. CXCR3^-^ B cells showed a distinct, partially overlapping set of significant genes in both compartments (**Supplementary Table 4**).

Given that the disease-associated signature was not restricted to CXCR3+ B cells, we asked whether CXCR3 positive and negative B cells differ from one another directly. Within PB, the Zn/MT signature was significantly higher in the positive cells in MS patients (paired Wilcoxon signed-rank p = 0.016, n = 18), but not in HC (p = 0.383, n = 8). This indicates a disease-specific divergence between these populations that is not apparent from the MS vs HC comparison alone. This pattern was absent in CSF (**Supplementary Figures S6**, **S7**). Five genes drove the PB effect, all more highly expressed in CXCR3+ cells in MS specifically: MT2A, MT1X, MT1F, MT1E, and SLC39A7 (ZIP7). Notably, three of these are MTs, whose expression goes in the opposite direction from the transporter-dominated MS vs HC signal, suggesting the CXCR3^+^-versus-CXCR3⁻ divergence and the MS vs HC disease signal may reflect at least partially distinct regulatory axes.

We found no evidence that CXCR3^+^ B cells reprogram Zn/MT expression upon moving from PB to CSF: neither individual genes nor the composite signature differed significantly between compartments, in either HC or MS (**Figure 3**; **Supplementary Figure 4**). A competitive gene-set rank test did show that this gene panel varies significantly less across the PB-to-CSF transition than the typical tested gene, in both HC and MS to a similar degree, consistent with zinc-handling genes being under tighter regulatory constraint than most transcripts generally, but this property was not disease-specific and should not be read as evidence of active compartment-specific reprogramming in CXCR3^+^ cells.

It is worth distinguishing two related but separate claims: the CSF-restricted detection of individual Zn/MT gene changes in CXCR3^+^ B cells (**Figure 2**; **Supplementary Table 3**) indicates that the MS-associated signal is more resolved in CSF than PB, whereas the competitive gene-set test (**Figure 3**) argues against active reprogramming of this gene set as CXCR3^+^ cells transition between compartments. The former reflects where the disease signature is statistically detectable; the latter addresses whether compartment entry itself drives the change. Our data support the first but not the second for the CXCR3^+^ subset.

In contrast, CXCR3⁻ B cells showed a clear compartment-specific shift in MS: seven of fourteen genes differed significantly between PB and CSF, with MTs (MT1X, MT1E, MT1F, MT2A) higher in CSF and transporters showing a mixed pattern, including SLC39A11 and SLC39A9 lower in CSF (**Supplementary Figure 5**, **Supplementary Table 5**). This pattern was absent in HC. Because this shift occurred in the CXCR3⁻, not the CXCR3^+^, population, and involved increased rather than decreased MT expression, it does not support a straightforward “reduced buffering, increased vulnerability” model for CXCR3^+^ effector cells entering the CNS. It may instead reflect a broader, disease-associated adaptation of the CSF B-cell compartment generally, distinct from CXCR3^+^-specific effector programming, and warrants further investigation with methods that can resolve additional B-cell subsets.

This study has several limitations. The number of patients and CSF B cells is modest, which reduces power for patient-level analyses and may contribute to the weak, non-significant correlations between zinc signatures and EDSS. Effect sizes for many Zn/MT genes are relatively small, so even statistically significant log2 fold-changes should be interpreted as subtle tuning of zinc handling rather than large transcriptional reprogramming. In addition, we rely on re-analysis of existing scRNA-seq datasets without direct measurements of zinc concentrations or protein expression in the same cells, and residual batch effects or annotation differences between studies cannot be fully excluded despite harmonization. Finally, these data are correlative; they support an association between B-cell states and altered zinc handling but do not establish whether zinc dysregulation is a driver or a consequence of CNS-directed autoimmunity in MS.

Together, these findings suggest that CXCR3^+^ and CXCR3⁻ B cells may acquire their disease-associated zinc-handling phenotypes at different points relative to CNS entry. The CXCR3^+^-specific elevation of MT and SLC39A7 (ZIP7) was detectable in blood, in MS but not HC, with no additional shift upon entry into CSF, consistent with this signature being established during peripheral activation or differentiation, rather than induced locally by the CSF microenvironment. CXCR3⁻ B cells showed the converse pattern: no disease-specific difference from CXCR3^+^ cells in blood, but a clear compartment-specific shift toward increased MT expression in CSF in MS.

This asymmetry could reflect two non-exclusive processes. First, CXCR3^+^ effector B cells may be zinc-reprogrammed as part of their peripheral activation, potentially linked to BCR or chemokine signaling known to mobilize intracellular zinc via ZIP transporters, and traffic to the CNS in an already-adapted state. Second, the CXCR3⁻ compartment shift may reflect local adaptation within the CSF, given that CSF B cells are not exclusively recent peripheral infiltrators: a substantial resident population can derive from meningeal and other CNS-border lymphoid niches and may be maintained or further differentiated locally rather than trafficking directly from blood (19, 23). Distinguishing between a purely infiltration-driven model and a locally adapted resident-population model, for instance, using approaches that separate recently infiltrated from CSF-resident B cells, or clonal lineage tracing between compartments, would help resolve whether the CXCR3⁻ shift reflects import of an already-changed population, *in situ* adaptation of resident cells, or both.

## Data Availability

Datasets analyzed in this study are available on the GEO repository website https://ncbi.nlm.nih.gov/geo. The code produced for this study is available here: https://github.com/RemiFritzen/CXCR3-BCell.
