# Supplementary material for: Zinc and metallothionein dysregulation in B cells in multiple sclerosis, with disease-specific CXCR3+/CXCR3⁻ divergence in blood
Source: Front Immunol. 2026 Aug 28;17:1922905. doi: 10.3389/fimmu.2026.1922905 (PMC13561944; doi:10.3389/fimmu.2026.1922905)
Supplement: Supplementary file 1 [file Supplementaryfile1.docx]

Supplementary Material

**Supplementary methods**

*Data integration and preprocessing*

Public single-cell datasets were obtained manually from their original repositories using txg and  fastq-dump , together with associated sample manifests and metadata files. Raw sequencing files were uploaded to the 10x Genomics cloud and processed using Cell Ranger version 10.0 to generate filtered count matrices. The resulting filtered output archives were then downloaded locally and converted to  .h5ad  objects using the custom 10xToh5ad.py  script included in the project repository. Downstream harmonisation and analysis were performed in Python using Scanpy-based workflows on the resulting AnnData objects.

*Batch integration.* Following normalization (total-count normalization to 10,000 counts per cell, log1p transformation) and selection of 2,000 highly variable genes (curated zinc transporter and metallothionein genes were retained regardless of variable-gene rank), datasets were integrated using Harmony (harmonypy 2.0.0), with dataset of origin as the batch covariate, applied to the first 30 principal components. The Harmony-corrected embedding was used for nearest-neighbor graph construction and UMAP visualization (Figure 1B). Harmony integration was not applied across tissue (PB/CSF) as a batch variable, since the CSF-associated B-cell cluster was determined to reflect genuine biological separation rather than a batch artifact.

*B-cell gate thresholds.* B cells were identified using a two-step gate: cells expressing any curated B-cell marker (raw count > 0) were retained, followed by exclusion of cells in the top 30% of mean non-B-marker expression among B-marker-positive cells, as likely contaminants or doublets. CXCR3⁺ cells were defined as any cell with detectable CXCR3 (UMI > 0); the distribution of CXCR3 expression did not show a clear bimodal separation supporting a higher threshold (Figure S1A–B).

*Zinc/metallothionein panel exclusions.* Four metallothionein genes (MT1A, MT1G, MT1H, MT1M) were excluded from the curated panel due to negligible expression (<1% of CXCR3⁺ cells with detectable transcript, in both PB and CSF; Table S2).

*Competitive gene-set rank test.* To detect coordinated shifts in the zinc/metallothionein panel that might not reach significance at the level of any single gene or the panel-average composite score, a competitive gene-set rank test was applied to each comparison's differential expression results. A location test compared the panel's log2 fold-change values against all other tested genes' log2 fold-change values (two-sided Mann–Whitney U); because this is a competitive test, a non-zero median log2FC in the background gene set was assessed alongside each result to distinguish an absolute shift in the panel from a relative deviation against a broader compositional pattern. A complementary, direction-agnostic magnitude test compared the panel's absolute log2 fold-change (|log2FC|) against background genes, testing whether the panel varies more or less than the typical tested gene, independent of direction.

*Dataset-of-origin confounding checks.* For unpaired comparisons (MS vs. HC), confounding was assessed via (i) the same test repeated within each source dataset separately, and (ii) a formal interaction test (OLS: signature ~ condition × dataset). For paired comparisons (CXCR3⁺ vs. CXCR3⁻; PB vs. CSF), confounding was assessed via (i) the paired test repeated within each dataset, and (ii) an unpaired Mann–Whitney U test comparing each patient's own paired difference between datasets, testing whether the magnitude of the within-patient shift itself differs by dataset of origin.

*Leave-one-out robustness.* For any gene–EDSS correlation surviving Benjamini–Hochberg correction, robustness was assessed by recomputing the Spearman correlation with each patient removed in turn, to verify the result was not driven by a single high-leverage observation.

*Effect-size magnitude.* Reported log2 fold-changes are smaller in magnitude than would be obtained from a naive per-cell calculation, for two reasons independent of the significance-testing correction above. First, patient-level pseudobulk aggregation weights every patient equally regardless of cell count, whereas a per-cell calculation is implicitly weighted by cells contributed per patient. Second, per-cell values are more sensitive to a small number of outlier cells (e.g., doublets, ambient RNA); collapsing to one value per patient before comparing groups averages out this cell-level noise, yielding a more conservative and biologically representative estimate of the population-level effect size.

*Software versions.* Python 3.12.11; numpy 2.4.6; pandas 3.0.5; scipy 1.18.0; scanpy 1.12.1; anndata 0.12.16; harmonypy 2.0.0; statsmodels 0.14.6; matplotlib 3.10.9; seaborn 0.13.2; PyYAML 6.0.3; h5py 3.16.0.

**Supplementary Tables**

**Table S1:** general characteristics of the cohorts from both datasets and number of cells observed in each compartment.

| Patient ID | Age | Sex (F%) | EDSS | Condition | CSF | | | PB | | |
| --- | --- | --- | --- | --- | --- | --- | --- | --- | --- | --- |
|  |  |  |  |  | #cells | #CXCR3+ | #CXCR3- | #cells | #CXCR3+ | #CXCR3- |
| **GSE138266** | 25-47 | 58 | 0.0-4.5 | Non-MS/MS | 3740 | 1206 | 2534 | 7762 | 435 | 7327 |
| MS19270 | 35 | F | 0.0 | MS | 650 | 217 | 433 | 1300 | 99 | 1201 |
| MS49131 | 47 | F | 4.5 | MS | 187 | 58 | 129 | 1106 | 108 | 998 |
| MS58637 | 22 | M | 0.0 | MS | 218 | 45 | 173 |  |  |  |
| MS60249 | 28 | F | 1.0 | MS | 1287 | 573 | 714 | 790 | 21 | 769 |
| MS71658 | 47 | F | 6.0 | MS | 318 | 75 | 243 | 585 | 47 | 538 |
| MS74594 | 42 | M | 0.0 | MS | 74 | 26 | 48 | 1473 | 93 | 1380 |
| PST83775 | 43 | F | - | MS | 548 | 129 | 419 | 645 | 16 | 629 |
| PST95809 | 43 | M | - | Non-MS | 108 | 20 | 88 | 290 | 12 | 278 |
| PTC32190 | 33 | M | - | Non-MS | 201 | 32 | 169 | 369 | 8 | 361 |
| PTC41540 | 32 | F | - | Non-MS | 76 | 15 | 61 | 978 | 20 | 958 |
| PTC85037 | 25 | F | - | Non-MS | 73 | 16 | 57 | 226 | 11 | 215 |
| **GSE133028** | 22-54 | 69 | 0.0-4.0 | HC/MS | 6015 | 1989 | 4026 | 21418 | 2311 | 19107 |
| 2 | 33 | M | - | HC | 64 | 26 | 38 | 1236 | 183 | 1053 |
| 3 | 27 | F | - | HC | 228 | 37 | 191 | 1303 | 180 | 1123 |
| 6 | 44 | M | 2.0 | MS | 383 | 81 | 302 | 1848 | 169 | 1679 |
| 7 | 37 | M | 1.5 | MS | 246 | 70 | 176 | 1175 | 108 | 1067 |
| 8 | 32 | M | 1.5 | MS | 587 | 244 | 343 | 330 | 88 | 242 |
| 9 | 27 | F | 3.5 | MS | 414 | 109 | 305 | 1066 | 26 | 1040 |
| 10 | 45 | F | 2.0 | MS | 267 | 128 | 139 | 1315 | 178 | 1137 |
| 16 | 22 | M | 4.0 | MS | 1149 | 361 | 788 | 2379 | 399 | 1980 |
| 22 | 35 | F | 4.0 | MS | 84 | 44 | 40 | 1705 | 76 | 1629 |
| 24 | 45 | F | 0.0 | MS | 330 | 88 | 242 | 2114 | 258 | 1856 |
| 25 | 42 | F | 1.5 | MS | 202 | 39 | 163 | 1341 | 104 | 1237 |
| 27 | 37 | F | 1.5 | MS | 330 | 88 | 242 | 899 | 103 | 796 |
| 28 | 50 | F | 4.0 | MS | 453 | 118 | 335 | 819 | 32 | 787 |
| 29 | 54 | M | 2.0 | MS | 228 | 37 | 191 | 1061 | 98 | 963 |
| 31 | 53 | F | 2.5 | MS | 719 | 405 | 314 | 1661 | 194 | 1467 |
| 32 | 41 | M | - | HC | 331 | 114 | 217 | 1166 | 115 | 1051 |

**Table S2: Zinc gene expression across compartments in CXCR3^+^ B cells.**

|  | CSF (3195 cells) | | PB (2746 cells) | |
| --- | --- | --- | --- | --- |
| gene | % expressing | mean count | % expressing | mean count |
| SLC39A1 | 11.96 | 0.1380 | 12.67 | 0.1486 |
| SLC39A4 | 8.61 | 0.0986 | 8.01 | 0.0914 |
| SLC39A6 | 11.71 | 0.1311 | 11.69 | 0.1373 |
| SLC39A7 | 12.27 | 0.1562 | 13.73 | 0.1770 |
| SLC39A9 | 9.67 | 0.1211 | 15.37 | 0.2021 |
| SLC39A10 | 21.82 | 0.3045 | 22.54 | 0.2877 |
| SLC39A11 | 16.09 | 0.2751 | 22.07 | 0.3922 |
| SLC39A14 | 2.13 | 0.0219 | 1.78 | 0.0211 |
| SLC30A1 | 4.10 | 0.0448 | 2.84 | 0.0299 |
| SLC30A9 | 15.84 | 0.2034 | 20.90 | 0.2695 |
| MT1E | 8.79 | 0.1264 | 8.96 | 0.2112 |
| MT1F | 13.05 | 0.1574 | 14.75 | 0.1970 |
| MT1X | 23.82 | 0.3433 | 23.96 | 0.3609 |
| MT2A | 42.00 | 1.1340 | 42.02 | 1.2465 |
| MT1A | 0.25 | 0.0025 | 0.04 | 0.0004 |
| MT1G | 0.53 | 0.0075 | 0.22 | 0.0040 |
| MT1H | 0.00 | 0.0000 | 0.04 | 0.0004 |
| MT1M | 0.13 | 0.0013 | 0.04 | 0.0004 |

**Table S3:** **Significantly differentially expressed genes between MS and HC subgroups in CXCR3⁺ B cells (CSF).** Wilcoxon rank-sum test on patient-level pseudobulk values; n = 8 HC, 19 MS patients; Benjamini–Hochberg correction shown genome-wide and restricted to the curated 14-gene zinc/metallothionein panel.

| Tissue | Gene | Log2FC | P_value_ | P_adj_ (genome-wide) | P_adj_(zinc pannel) |
| --- | --- | --- | --- | --- | --- |
| CSF | SLC30A1 | 0.036 | 1.09.10^-2^ | 9.67.10^-2^ | 2.55.10^-2^ |
| CSF | SLC30A9 | 0.158 | 1.43.10^-3^ | 5.32.10^-2^ | 1.40.10^-2^ |
| CSF | SLC39A6 | 0.070 | 6.75.10^-3^ | 7.16.10^-2^ | 1.89.10^-2^ |
| CSF | SLC39A9 | 0.105 | 2.95.10^-3^ | 5.32.10^-2^ | 1.40.10^-2^ |
| CSF | SLC39A11 | 0.186 | 4.01.10^-3^ | 5.32.10^-2^ | 1.40.10^-2^ |
| CSF | SLC39A14 | 0.024 | 3.84.10^-3^ | 5.32.10^-2^ | 1.40.10^-2^ |

**Table S4:** **Significantly differentially expressed genes between MS and HC in CXCR3⁻ B cells**. Wilcoxon rank-sum test on patient-level pseudobulk values, unpaired; Benjamini–Hochberg correction shown genome-wide and restricted to the curated 14-gene zinc/metallothionein panel.

| Tissue | Gene | Log2FC | P_value_ | P_adj_ (genome-wide) | P_adj_(zinc pannel) |
| --- | --- | --- | --- | --- | --- |
| PB | SLC30A9 | 0.136 | 3.35E-03 | 9.83E-02 | 1.56E-02 |
| PB | SLC39A6 | 0.036 | 1.58E-02 | 1.62E-01 | 3.69E-02 |
| PB | SLC39A9 | 0.110 | 1.02E-03 | 8.96E-02 | 1.42E-02 |
| PB | SLC39A10 | 0.142 | 1.33E-02 | 1.62E-01 | 3.69E-02 |
| PB | SLC39A11 | 0.234 | 7.63E-03 | 1.34E-01 | 2.67E-02 |
| PB | SLC39A14 | 0.009 | 2.91E-03 | 9.83E-02 | 1.56E-02 |
| CSF | MT1X | -0.185 | 7.91E-03 | 1.00E-01 | 3.69E-02 |
| CSF | SLC30A9 | 0.127 | 7.91E-03 | 1.00E-01 | 3.69E-02 |
| CSF | SLC39A6 | 0.043 | 7.91E-03 | 1.00E-01 | 3.69E-02 |

**Table S5: Significantly differentially expressed genes between CSF *vs.* PB in CXCR3⁻ B cells from MS patients**. Wilcoxon signed-rank test on paired patient-level pseudobulk values (n = 18 patient pairs); Benjamini–Hochberg correction shown genome-wide and restricted to the curated 14-gene zinc/metallothionein panel. No genes reached significance in HC (n = 8 patient pairs; closest: padj = 0.128) — HC results not tabulated.

| Condition | Gene | Log2FC | P_value_ | P_adj_ (genome-wide) | P_adj_(zinc pannel) |
| --- | --- | --- | --- | --- | --- |
| MS | MT1E | 0.056 | 4.20E-04 | 1.05E-03 | 2.94E-03 |
| MS | MT1F | 0.048 | 3.36E-03 | 5.71E-03 | 1.40E-02 |
| MS | MT1X | 0.123 | 1.53E-05 | 7.11E-05 | 2.14E-04 |
| MS | MT2A | 0.136 | 1.20E-02 | 1.83E-02 | 2.81E-02 |
| MS | SLC39A9 | -0.037 | 4.01E-03 | 6.66E-03 | 1.40E-02 |
| MS | SLC39A10 | 0.064 | 1.82E-02 | 2.61E-02 | 3.65E-02 |
| MS | SLC39A11 | -0.111 | 8.96E-03 | 1.42E-02 | 2.51E-02 |

**Table S6: Leave-one-out (LOO) robustness check for the SLC39A7-EDSS correlation in PB**

| Dropped patient | Rho | P-value |
| --- | --- | --- |
| 10 | 0.712 | 1.3E-03 |
| 16 | 0.728 | 9.1E-04 |
| 22 | 0.847 | 1.8E-05 |
| 24 | 0.743 | 6.3E-04 |
| 25 | 0.772 | 2.9E-04 |
| 27 | 0.697 | 1.9E-03 |
| 28 | 0.691 | 2.1E-03 |
| 29 | 0.712 | 1.3E-03 |
| 31 | 0.714 | 1.3E-03 |
| 6 | 0.712 | 1.3E-03 |
| 7 | 0.720 | 1.1E-03 |
| 8 | 0.720 | 1.1E-03 |
| 9 | 0.732 | 8.3E-04 |
| MS19270 | 0.686 | 2.4E-03 |
| MS49131 | 0.714 | 1.3E-03 |
| MS60249 | 0.676 | 2.9E-03 |
| MS71658 | 0.671 | 3.2E-03 |
| MS74594 | 0.680 | 2.7E-03 |

**Supplementary Figures**

**Figure S1: CXCR3 expression in collated GSE138266 and GSE133028 datasets and quality control checks.** (A) Histogram of CXCR3 UMI counts across B cells, showing that most cells have low or undetectable CXCR3 expression. (B) Histogram of log1-transformed CXCR3 UMI counts among detected cells only, highlighting the distribution of signal in the CXCR3-positive subset. (C) Violin plots comparing the distribution of total UMI counts per cell between the CSF and PB samples. Individual cells are shown as points, with the median and interquartile range overlaid. (D) Violin plots showing the percentage of counts assigned to mitochondrial genes per cell in the CSF and PB samples. Individual cells are shown as points, with summary statistics overlaid. (E) Total number of cells retained after quality control for each sample (CFP and PB).

**Figure S2:** Volcano plot of differential gene expression between multiple sclerosis (MS) and healthy controls (HC) in (A) peripheral blood (PB) CXCR3⁺ B cells and (B) CSF. The x‑axis shows log₂ fold‑change (MS vs HC) and the y‑axis shows −log₁₀(p_adj_). Zinc/metallothionein (Zinc/MT) genes are highlighted, with significantly altered genes (p_adj_ < 0.05) in color and non‑significant Zinc/MT genes and all other genes shown as indicated. (B) Patient-level zinc/metallothionein signature in PB CXCR3⁺ B cells, showing higher signature values in MS than healthy controls (Mann–Whitney p ≈ 2.85.10^⁻1^). (D) Patient-level zinc/metallothionein unsigned signature in CSF CXCR3⁺ B cells (Mann–Whitney p ≈ 4.94.10^⁻2^).

**Figure S3:** **Zinc/metallothionein genes and signature in CXCR3^-^ B cells from HC vs MS in the PB and CSF.** (A) Log2 fold‑change (MS vs healthy controls) of zinc transporter and metallothionein genes in peripheral blood (PB) CXCR3⁺ B cells, highlighting upregulation of *SLC39A11* and several zinc transporters in MS. (B) Log2 fold‑change (MS vs healthy controls) of zinc/metallothionein genes in cerebrospinal fluid (CSF) CXCR3^-^ B cells, with multiple transporters increased and metallothioneins reduced in MS. Violin plots of zinc/metallothionein signature in (C) PB and (D) CSF CXCR3^-^ B cells. The statistical test is made on a per-patient average of signature zinc values in MS and healthy controls PB (Mann–Whitney p ≈ 4.16.10⁻^3^) and CSF (Mann–Whitney p ≈ 5.55.10⁻^4^)

**Figure S4: Compartmental differences in zinc/metallothionein genes and signature in CXCR3⁺ B cells.** (A) Volcano plot of differential gene expression (CSF vs PB) in CXCR3⁺ B cells from MS patients. The x-axis shows log₂ fold-change (CSF vs PB) and the y-axis shows −log₁₀(padj); curated zinc transporter and metallothionein genes are highlighted, with all other genes shown in grey. No gene reached significance. (B) Log₂ fold-change (CSF vs PB) of the same curated zinc/metallothionein genes in CXCR3⁺ B cells from MS patients, ranked by effect size. Several transporters (*SLC39A9*, *SLC39A11*, *SLC39A7*) trend lower in CSF relative to PB, but none reached significance after correction. (C) Volcano plot of differential gene expression (CSF vs PB) in CXCR3⁺ B cells from healthy controls (HC), as in (A). No gene reached significance. (D) Log₂ fold-change (CSF vs PB) of the same genes in CXCR3⁺ B cells from HC, ranked by effect size. *MT2A* is modestly enriched in CSF, whereas most transporters trend higher in PB, though none reached significance after correction. (E) Patient-level zinc/metallothionein (Zinc/MT) signature (unsigned) in HC CXCR3⁺ B cells, comparing PB and CSF. Each point represents one patient; boxes show the median and interquartile range, and lines connect each patient's own PB and CSF values. The composite signature did not differ significantly between compartments (paired Wilcoxon signed-rank p = 1.21 × 10⁻¹, n = 7 patients). (F) As in (E), for CXCR3⁺ B cells from MS patients. The composite signature did not differ significantly between compartments (paired Wilcoxon signed-rank p = 2.01 × 10⁻¹, n = 18 patients).

**Figure S5: Compartmental differences in zinc/metallothionein genes and signature in CXCR3⁻ B cells.** (A) Volcano plot of differential gene expression (CSF vs PB) in CXCR3⁻ B cells from MS patients. The x-axis shows log₂ fold-change (CSF vs PB) and the y-axis shows −log₁₀(padj); curated zinc transporter and metallothionein genes are highlighted, with genes reaching significance after Benjamini–Hochberg correction restricted to the curated panel (padj < 0.05) shown in a distinct color. Seven of fourteen curated genes reached significance. (B) Log₂ fold-change (CSF vs PB) of the same curated zinc/metallothionein genes in CXCR3⁻ B cells from MS patients, ranked by effect size; asterisks indicate significance after panel-restricted correction. *MT1X*, *MT1E*, *MT1F*, and *MT2A* were higher in CSF, while *SLC39A11*, *SLC39A9*, and *SLC39A10* showed a mixed pattern among the transporters. (C) Volcano plot of differential gene expression (CSF vs PB) in CXCR3⁻ B cells from healthy controls (HC), as in (A). No gene reached significance. (D) Log₂ fold-change (CSF vs PB) of the same genes in CXCR3⁻ B cells from HC, ranked by effect size; no gene reached significance. (E) Patient-level zinc/metallothionein (Zinc/MT) signature (unsigned) in HC CXCR3⁻ B cells, comparing PB and CSF. Each point represents one patient; boxes show the median and interquartile range, and lines connect each patient's own PB and CSF values. The composite signature did not differ significantly between compartments (paired Wilcoxon signed-rank p = 0.109, n = 7 patients). (F) As in (E), for CXCR3⁻ B cells from MS patients. The composite signature did not differ significantly between compartments (paired Wilcoxon signed-rank p = 0.181, n = 18 patients).

**Figure S6: Paired comparison of CXCR3⁺ and CXCR3⁻ B cells in peripheral blood.** (A) Volcano plot of differential gene expression (CXCR3⁺ vs CXCR3⁻) in PB B cells from MS patients, paired at the patient level. Curated zinc transporter and metallothionein genes are highlighted; filled circles indicate genes reaching significance after Benjamini–Hochberg correction restricted to the curated panel (padj < 0.05). (B) Log2 fold-change of zinc/metallothionein genes (CXCR3⁺ vs CXCR3⁻) in PB B cells from MS patients, ranked by effect size; asterisks indicate significance after panel-restricted correction (*padj < 0.05, **padj < 0.01, ***padj < 0.001). Five genes (MT2A, MT1X, MT1F, MT1E, SLC39A7) were significantly higher in CXCR3⁺ B cells. (C, D) As in (A, B), for HC PB B cells; no gene reached significance. (E, F) Paired patient-level composite zinc/metallothionein signature (sign-corrected) for CXCR3⁻ and CXCR3⁺ PB B cells in HC (E) and MS (F); each line connects one patient's own CXCR3⁻ and CXCR3⁺ values. The signature was significantly higher in CXCR3⁺ B cells in MS (paired Wilcoxon signed-rank p = 0.016, n = 18 patients) but not in HC (p = 0.383, n = 8).

**Figure S7. Paired comparison of CXCR3⁺ and CXCR3⁻ B cells in cerebrospinal fluid.** (A) Volcano plot of differential gene expression (CXCR3⁺ vs CXCR3⁻) in CSF B cells from MS patients, paired at the patient level, as in Figure S6A. (B) Log2 fold-change of zinc/metallothionein genes (CXCR3⁺ vs CXCR3⁻) in CSF B cells from MS patients, ranked by effect size. (C, D) As in (A, B), for HC CSF B cells. (E, F) Paired patient-level composite zinc/metallothionein signature (sign-corrected) for CXCR3⁻ and CXCR3⁺ CSF B cells in HC (E) and MS (F), as in Figure S6E-F. Unlike in PB, the composite signature did not differ significantly between CXCR3⁻ and CXCR3⁺ B cells in CSF in either MS (paired Wilcoxon signed-rank p = 0.717, n = 19 patients) or HC (p = 0.547, n = 8). Unlike in PB (Table S6), no gene reached significance in CSF after correction, in either HC or MS (closest: SLC39A11, padj = 0.053 in MS).

**Figure S8: Individual gene expression plotted against each patient’s expanded disability status scale (EDSS).** Scatter plots showing mean signed, log‑normalised expression of zinc genes in cerebrospinal fluid (CSF, left) and peripheral blood (PB, right). B cells plotted against EDSS for individual patients (N = 18). Each point represents one patient and is annotated with the patient identifier. Dashed lines show linear regression fits with Spearman correlation coefficients and p values indicated in the panel titles. Only *SLC39A7* in PB showed an association with EDSS (p_adj_ : 0.021). The robustness of this result was assessed by testing the strength of the association when taking one patient at a time (leave one out (LOO – Table S6), which shows that no single patient is driving the significant difference.
